# A Facile Approach for Rapid Prototyping of Microneedle Molds, Microwells and Micro-Through-Holes in Various Substrate Materials Using CO_2_ Laser Drilling

**DOI:** 10.3390/biomedicines8100427

**Published:** 2020-10-18

**Authors:** Yu-Wei Chen, Mei-Chin Chen, Kuang-Wei Wu, Ting-Yuan Tu

**Affiliations:** 1 Department of Biomedical Engineering, National Cheng Kung University, Tainan 70101, Taiwan; edfu237bc@gmail.com (Y.-W.C.); stephen0001345@gmail.com (K.-W.W.); 2 Department of Chemical Engineering, National Cheng Kung University, Tainan 70101, Taiwan; kokola@mail.ncku.edu.tw; 3 Medical Device Innovation Center, National Cheng Kung University, Tainan 70101, Taiwan; 4 International Center for Wound Repair and Regeneration, National Cheng Kung University, Tainan 70101, Taiwan

**Keywords:** CO_2_ laser, rapid prototyping, microneedle, multicellular tumor spheroids, microwells, hanging drops

## Abstract

CO_2_ laser manufacturing has served as an enabling and reliable tool for rapid and cost-effective microfabrication over the past few decades. While a wide range of industrial and biological applications have been studied, the choice of materials fabricated across various laser parameters and systems is often confounded by their complex combinations. We herein presented a unified procedure performed using percussion CO_2_ laser drilling with a range of laser parameters, substrate materials and various generated microstructures, enabling a variety of downstream tissue/cellular-based applications. Emphasis is placed on delineating the laser drilling effect on different biocompatible materials and proof-of-concept utilities. First, a polydimethylsiloxane (PDMS) microneedle (MN) array mold is fabricated to generate dissolvable polyvinylpyrrolidone/polyvinyl alcohol (PVP/PVA) MNs for transdermal drug delivery. Second, polystyrene (PS) microwells are optimized in a compact array for the formation of size-controlled multicellular tumor spheroids (MCTSs). Third, coverglass is perforated to form a microaperture that can be used to trap/position cells/spheroids. Fourth, the creation of through-holes in PS is validated as an accessible method to create channels that facilitate medium exchange in hanging drop arrays and as a conducive tool for the growth and drug screenings of MCTSs.

## 1. Introduction

Microfabrication techniques have revolutionized the way biologists and medical scientists conduct studies in the last few decades [1]. The well-developed semiconductor workflow enables not only the creation of physical microstructures conducive to the dimension of cells and tissues but also integrates these structures for various downstream functional assays [2]. However, although microfabrication techniques have been widely applied in various areas of biomedical research, the dependence on lithographic procedures that require either chemical etching with special equipment and cleanroom facilities or a silicon master for replica molding has been criticized as a barrier to entry. Even minor changes in the design of a microstructure require a cumbersome and laborious process accompanied by a significant increase in the fabrication and materials cost [3]. Although most biomedical laboratories can utilize alternative commercial products with relative ease, the lack of flexibility in changing the design and protocol integration as well as the costly nature often hamper the utility of these alternatives. Identifying a rapid and economical method for the reliable generation of microstructures has thus become crucial, especially in terms of methods that allow for quick iterations in design modification during initial stages of validation, which is imperative for individual labs.

Laser machining has long been utilized as a salient alternative or supplementary tool to some lithography-based approaches for microfabrication since the late 1980s [3,4]. CO_2_ lasers are one of the most conventionally used laser sources for rapid prototyping and cost-effective microfabrication. Microchannel engraving has been demonstrated as a rapid fabrication method for microfluidic devices [5,6]. Laser drilling, termed ablation, of a single spot is another conventional technique to generate an array of microstructures, i.e., concavities or through-holes applied in many industrial and research applications [7,8]. Combined with the use of different substrate materials, a wide range of downstream tissue or cellular-based utilities can be readily achieved [3,9,10,11]. For example, microscale needles, termed microneedles (MNs), enable the delivery of large molecules, such as proteins, DNA and vaccines, into the skin in a painless, rapid and efficient way [12]. The female MN polydimethylsiloxane (PDMS) mold, used for fabrication of polymeric MNs, can be easily and conveniently made by laser drilling. Additionally, microwell technology fabricated by CO_2_ lasers has been applied in generating cellular spheroids [3,13] and circulating tumor cell cultures [14]. Microwells are conducive to the formation of uniform, size-controlled, multicellular tumor spheroids (MCTSs). MCTSs imitate three-dimensional (3D) growth in a way similar to avascular tumors in vivo in terms of their cell–cell interaction. Studies have also indicated that drug screening carried out on MCTSs is more relevant than that of traditional two-dimensional (2D) cultures, suggesting the potential use of microwells in future drug screening and exploration of different cancer treatment modalities [15].

However, a major barrier to fabricating microstructure arrays using CO_2_ laser drilling is that different studies have only been reported sporadically, and these studies have involved different systems, substrate materials, and applications [3,8,9,16,17]. On the one hand, these outcomes have prohibited this technique from reaching the greater scientific community because of the concern that specific and unique protocols as well as meticulous pre/postmaterial processes are required for different applications. On the other hand, varying microstructure geometries and qualities from different studies [13,14,16] suggest ample room for further improvement based on a comprehensive and systematic understanding of the choice of both the materials and the laser system. With these notions, we hope to generate a holistic viewpoint of the simplicity and consistency of the method as well as of the diverse microstructures and applications that can be achieved through the integration of various common laboratory-accessible consumables.

**Figure 1:**
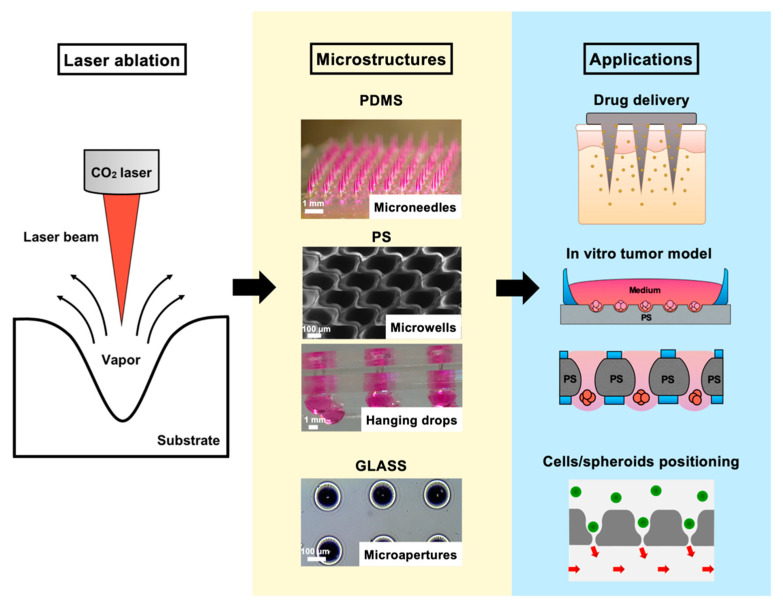
Illustration and images of the rapid laser drilling of different biocompatible materials for various downstream applications, including polydimethylsiloxane (PDMS) microneedle (MN) array molds for transdermal drug delivery; PS microwell/hanging drop arrays for 3D cell culture of in vitro tumor models, and glass macroapertures for trapping/positioning cells/spheroids (green) by a suction force (red arrows).

In this report, we present a facile laser ablation technique using a laboratory-built CO_2_ laser drilling system that can be conveniently applied to fabricate different biocompatible materials for generating arrayed microstructures. The primary goal was to establish a set of protocols that are similar in laser parameters and also applicable to various downstream cellular-based and drug discovery applications (Figure 1). Detailed laser parameters were inspected using different pulse numbers for single-dot laser drilling. PDMS was first assessed for its ability to form different MN mold geometries, and polyvinylpyrrolidone/polyvinyl alcohol (PVP/PVA) were employed to cast an MN array patch for transdermal drug delivery. Polystyrene (PS) microwells were optimized in an arrangement to better prevent cell loss during cell seeding steps and to form size-controlled MCTSs. Penetrated glass microapertures were assessed for their ability to capture MCTSs by applying negative suction. PS was also validated as a substrate to perforate a through-hole that was advantageous for media exchange in the hanging drop method for large MCTSs. The proposed methodology highlights a highly versatile and facile microfabrication technique synergizing the conventional CO_2_ laser and several biocompatible materials for a wide range of downstream applications.

## 2. Experimental Section

### 2.1. Laser Setup and Substrate Materials

A Synrad 48-1 CO_2_ laser (10 W, Synrad, Inc., Mukilteo, WA, USA) with a 10.6 µm wavelength was horizontally mounted on an adjustable frame that allowed adjustment along the z-axis. The diameter of the laser beam (3.5 mm) was broadened by a 2× beam expander and then focused by a plano-convex lens (2”). Two step motor X–Y stages positioned the substrate materials at the location of interest for laser ablation. The parts mentioned above were assembled by Laser Solution Technology Inc. (Taipei, Taiwan) based on the author’s conceptual design. Laser pulse commends were operated under pulse width modulations, in which the duty cycle was set at 60% duty at 5 kHz and the power was determined based on the number of pulses ablated on the choice of the materials. PDMS was fabricated by mixing elastomer (Sylgard-184A, Dow Corning Corp., Midland, MI, USA) and curing agent (Sylgard-184B, Dow Corning Corp.) at a weight ratio of 10 to 1. PS microscope slides (1 mm thick) were purchased from EMS (Electron Microscopy Sciences Inc., Hatfield, PA, USA). Commercial No.1 borosilicate coverglass (22 × 22 mm, Paul Marienfeld GmbH and Co. KG., Lauda-Königshofen, BW, Germany) was used as the glass substrate. Poly(methyl methacrylate) (PMMA) slides (1 mm thick) were acquired from a local manufacturer (Yi-Shiou Co. Ltd., Tainan, Taiwan).

### 2.2. Cell Culture

Human hepatocellular carcinoma cell lines, HepG2 and Huh7, were cultured at 37 °C in a 5% CO_2_ humidified incubator and maintained in high-glucose Dulbecco’s Modified Eagle’s Medium (DMEM, Sigma-Aldrich Corp., St. Louis, MO, USA) supplemented with 1% penicillin/streptomycin (Sigma-Aldrich Corp.) and 10% fetal bovine serum (FBS, Gibco, Thermo Fisher Scientific Co. Ltd., Waltham, MA, USA). Cultured cells were monitored daily and the supernatants were replaced with fresh medium every two or three days or at approximately 80–90% confluency.

### 2.3. MN Fabrication

A 50 wt% poly(vinyl pyrrolidone) (PVP, molecular weight (MW) 10,000 kDa, Sigma-Aldrich Corp.)/poly(vinyl alcohol) (PVA, MW 6000 kDa, Polysciences, Inc., Warrington, PA, USA) aqueous solution was prepared at a weight ratio of 1:1. PDMS molds were made by direct laser ablation on PDMS. A 30 W plasma treatment (Harrick Plasma Inc., Ithaca, NY, USA) was performed on the surface of the PDMS mold for 30 s. Tetramethylrhodamine isothiocyanate–dextran (TRITC-dextran, MW 155 kDa, Sigma-Aldrich Corp.) was added to deionized water and stirred until completely dissolved. The prepared PVP/PVA solution was mixed well with the TRITC-dextran aqueous solution to obtain a homogeneous solution containing 40 wt% PVP/PVA. As a first layer, the TRITC-dextran-containing PVP/PVA solution was added to the PDMS mold surface under a vacuum to ensure the complete filling of the MN mold cavities by the solution. Residual solution that did not enter into the mold cavities was removed from the mold surface. Under the same vacuum operation, the PVP/PVA solution without TRITC-dextran was then added to the mold surface as a second layer to constitute the patch. The filled molds were placed at room temperature for 30 min and dried in an oven at 37 °C for at least one day. The PVP/PVA MN array was then gently peeled from the mold for skin insertion tests.

### 2.4. MN Skin Insertion

To evaluate the MN skin insertion ability, the fabricated MNs were inserted into fully thick porcine skin with a thickness of 1186 ± 136 μm (*n* = 5) by using a custom-made applicator for 10 min. The applicator device provided a consistent force for MN application and ensured consistent MN penetration. After the complete dissolution of MNs in the skin, the MN-treated site was excised for histological examination.

### 2.5. PDMS Chamber Fabrication

PDMS chambers were made for both microwell and hanging drop applications. The PS slide was cleaned with 75% ethanol and air-dried, then treated with 30 W air plasma cleaner for 30 s and placed in an aqueous solution of 1% volume(v)/v (3-aminopropyl)triethoxysilane (APTES, Sigma-Aldrich Corp.) for at least 20 min. The slides were then washed with deionized water and air-dried. PDMS was also treated with air plasma under the same conditions used for the PS slide. Activated slides and PDMS were kept in conformal contact at 70 °C in an oven for 1 h.

### 2.6. MCTS Formation in Microwells

Microwells were first soaked within 75% ethanol to remove debris from the laser ablation and for disinfection. The microwells were then placed under irradiation with UV light for 30 min. Before cell seeding, microwells were coated with 0.2% Pluronic F-127 (Sigma-Aldrich Corp.) in 1× PBS for 30 min to prevent cell attachment to the PS substrate and then washed twice with 1× PBS. Then, the desired cell suspension concentrations (50, 100, 150, 200 cells microwell^−1^) of both Huh7 and HepG2 cells were loaded into the chamber. Cells were maintained at 37 °C in a 5% CO_2_ humidified incubator for 4 days, and the aggregation of the cells was recorded daily. Formed MCTSs were harvested by pipetting the medium twice to flush them from the microwells and then transferred to the container of interest for different downstream applications.

### 2.7. MCTS Trapping

The Huh-7 MCTS suspension solution was diluted to 300 MCTSs suspended in serum-free medium (50 µL). Hoechst solution (1:2000, Invitrogen, Thermo Fisher Scientific Co. Ltd.) was added to the cell suspension, and MCTSs were then incubated for 30 min at 37 °C. A PDMS chamber was made and sealed by a coverglass attachment. The cell suspension was then added to the top of the coverglass, and suction was applied to the sealed PDMS chamber by syringe (1 mL). The MCTCs were then trapped within an array of funnel-like glass apertures, and excessive MCTSs were removed by washing with 1× PBS.

### 2.8. MCTS Formation by the Hanging Drop Method

After disinfection with 75% ethanol spray, hybrid devices for use with the hanging drop method were exposed to UV light for 30 min for sanitizing and drying. The device was then placed on a bracing frame of sterilized PDMS in a culture dish (150 mm), and 0.2% Pluronic F-127 in 1× PBS (50 µL) was loaded into both sides of the PDMS wells for 30 min. Then, the Pluronic F-127 was aspirated, and the device was kept dry for further usage. After dilution in culture medium to obtain desired cell densities, cell solutions (20 µL) with specific cell numbers (500, 1000, 5000 cells droplet^−1^) were dispensed into the bottom side of the PDMS wells of each device (after inverting the device). Next, medium (50 μL) was added to the top side of the through-hole well to promote fluid exchange to the bottom side by gravity. Before maintaining the cultures at 37 °C in a 5% CO_2_ humidified incubator, the device was placed into a 100 mm culture dish that also contained sterilized ultrapure water (2 mL) in a culture dish (30 mm), which significantly reduced the rate of evaporation within the incubator. Owing to gravity, cells within hanging drops settled at the nadir of the medium/air interface at the bottom of the droplets and developed into a 3D aggregates or microtissue structures spontaneously during the 4 days of formation. Note that fresh culture medium exchange was performed daily, and we also ensured that the droplet structures on the bottom side of the PDMS wells were intact every day.

### 2.9. MCTS Anticancer Drug Screening and Viability Analysis

Doxorubicin hydrochloride (DOX, Sigma-Aldrich Corp.) at 5 mg mL^−1^ in DMSO was diluted with culture medium to 3.16, 10, 31.6, and 100 μM, dispensed into the hanging drops after 3 days of MCTS development, and then treated for 24 h. The morphology and viability of the MCTSs were monitored using a LIVE/DEAD™ Viability/Cytotoxicity Kit (Invitrogen, Thermo Fisher Scientific Co. Ltd.), where the calcein-AM and ethidium homodimer-1 were added at dilutions of 1:2000 and 1:1000, respectively. After incubation at 37 °C in a 5% CO_2_ humidified incubator for 1 h, MCTSs were recorded under an inverted fluorescence microscope. The quantitative viability measurement of MCTSs in hanging drops was also assessed using a WST-1 Cell Proliferation Assay Kit (Takara Bio Inc., Kusatsu, Shiga, Japan), in which WST-1 reagent (10 μL) was added to culture medium (100 μL) containing a spheroid in each drop, followed by transfer to a 96-well plate. After incubation for 2 h in the incubator, the absorbance at 450 nm was measured using a spectrophotometer (Hitachi Ltd., Tokyo, Japan).

### 2.10. Statistical Analysis

Numerical values of dimensions in our study were expressed as the means ± standard deviations of more than three independent replicates. Statistical significance was determined via one-way analysis of variance (ANOVA) with Prism 7 (GraphPad Software Inc., San Diego, CA, USA). Statements of significance were based on *p*-values < 0.05.

## 3. Results

### 3.1. Creation of Microstructured PDMS Molds for Fabrication of Dissolving PVP/PVA MN Array

**Figure 2:**
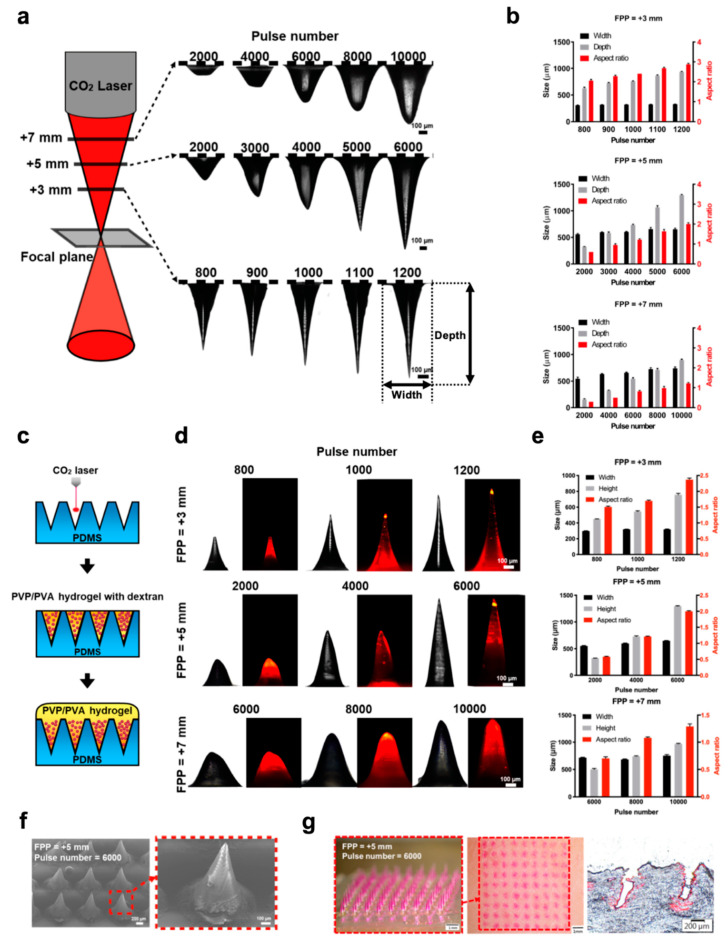
Fabrication of polyvinylpyrrolidone/polyvinyl alcohol (PVP/PVA) MN arrays using a laser-ablated PDMS mold. (**a**) Schematic diagram of the laser-ablation process for creating PDMS molds with various CO_2_ laser pulse numbers in focal plane positions (FPPs). (**b**) Evaluation of the widths, depths, and aspect ratios of the PDMS molds created with different pulse numbers and FPPs. (**c**) Schematic diagrams of PVP/PVA MN fabrication from ablated PDMS molds. (**d**) Characteristics and (**e**) specifications of Tetramethylrhodamine isothiocyanate TRITC-dextran-loaded PVP/PVA MNs. (**f**) SEM image of the PVP/PVA MN array. (**g**) Brightfield micrographs of PVP/PVA MNs containing TRITC-dextran (left), porcine cadaver skin after MN insertion (middle), and a corresponding histological section (right).

Here, we first used the CO_2_ laser ablation technique to fabricate PDMS molds for the production of polymeric MNs (Figure 2). To investigate the effect of the laser parameters on the shape and size of the created PDMS mold, PDMS slabs were first placed at focal plane positions (FPPs) of +3, +5, or +7 mm and various pulse numbers were applied to the sample (Figure 2a). We found that as the FPP decreased, the cross-sectional profile of the laser-ablated microcavities in the PDMS changed from U-shaped with blunt bottoms to V-shaped with sharp tips. The depth and aspect ratio of the ablated microcavities notably increased with an increase in laser pulse number. However, the width of the cavity did not change with the pulse number but did slightly increase with increased FPP. The effect of these parameters on the width, depth, and aspect ratio of the laser-ablated PDMS mold is quantified in Figure 2b. These results show that microcavities with different geometries and specifications can be created in the PDMS mold by simply adjusting the laser parameters.

We next fabricated polymeric MN arrays by casting PVP/PVA solutions into the mold (Figure 2c). Figure 2d,e display the characteristics and sizes of the PVP/PVA MNs prepared from the PDMS molds with different laser parameters. The tip curvature radii of all prepared MNs are shown in Figure S1 (Supporting Information). As shown, TRITC-dextran-loaded PVP/PVA MNs with various specifications were successfully fabricated from the molds using a vacuum-assisted micromolding process. Many factors affect the mechanical strength of MNs, including the material composition, geometry, aspect ratio, and tip curvature radius. According to our previous study [18], polymeric MNs with an aspect ratio of 2 exhibited good mechanical strength and skin insertion capability. Therefore, PVP/PVA MNs with a height of 1296 ± 8 μm, a base width of 649 ± 5 μm, and a tip curvature radius of 9 ± 1 μm (*n* = 6 needles), which were fabricated from the mold at a pulse of 6000 and an FPP of +5 mm, were used for skin insertion tests. Scanning electron microscopy (SEM) images showed that the prepared MN array had a sharp tip and a smooth surface (Figure 2f). To evaluate the potential of using the PVP/PVA MNs for transdermal drug delivery, TRITC-dextran was loaded in the MNs to serve as a model drug, and then the MNs were manually applied to a porcine skin. After insertion for 5 min, we observed that the PVP/PVA MNs were quickly dissolved in the skin, and a complete array of red spots (9 × 9) was visible on the skin surface, indicating that all of the PVP/PVA MNs were inserted into the skin (Figure 2g). Histological sections of the skin showed that the loaded drug (red) was successfully released from the MNs and delivered into the skin, and the MN penetration depth was 606 ± 47 μm (*n* = 5).

### 3.2. PS Microwells for the Formation of MCTS

**Figure 3:**
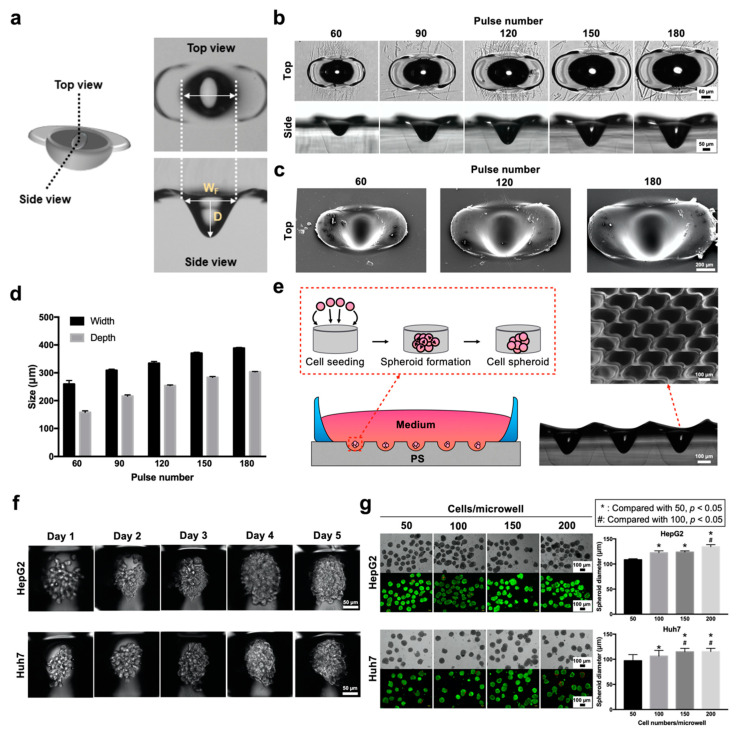
Characterization of polystyrene (PS) microwells made with different pulse numbers and multicellular tumor spheroid (MCTS) formation. (**a**) Illustration of ablated microwells from top and side views. (**b**) Top and side views of a single microwell with respect to pulse numbers ranging from 60 to 180. (**c**) A detailed surface profile of a single microwell recorded by SEM. (**d**) Evaluation of the widths and depths of microwells made with different pulse numbers. (**e**) Schematic diagram of spheroid formation within microwells, and a detailed profile of the microwell arrangement for spheroid formation by SEM. (**f**) MCTSs are directly shown from day 1, the day of cell seeding, to day 5 within the microwell. (**g**) Brightfield (gray) and fluorescent (live (green) /dead (red) staining) images of MCTSs with different cell number aggregates (50, 100, 150, 200 cells microwell^−1^) (left), followed by the quantification of spheroid size with respect to the cell seeding number (per microwells) (right).

Next, laser drilling for microwell generation was demonstrated for the formation of MCTSs using PS (Figure 3). The PS substrate was first ablated in a fashion similar to that used to make the MN mold (i.e., CO_2_ laser drilling with pulse numbers ranging from 60 to 180). The top view demonstrates that the ablation resulted in a concave geometry with a recast zone observed as a wing structure around its edge (Figure 3a). The side view captures the concave microwell structure, suggesting that the microwell possessed a smooth curvature formed on the bottom. Both the width and depth of the PS microwells grew in size as the number of laser pulses increased (Figure 3b). SEM images show a clean, concave curved bottom with smooth surface characteristics, and the recast PS protrudes at the edge (Figure 3c). The PS microwells were characterized by their width and depth (Figure 3d), which showed similar trends, increasing from 260–350 μm (width) and 180–280 μm (depth) as the laser pulse increased.

Given that the substrate was treated with cell attachment repellent, cells could aggregate in spheroids after 3 to 5 days, as shown in the schematic illustration (Figure 3e). To demonstrate that the microwells were highly consistent in their geometry and ability to generate MCTSs via varying cell seeding densities, 180 laser pulses were selected to fabricate the microwell array. The close-up SEM images demonstrate the staggered arrangement of the microwell array, with the recasting zones overlaying each other. This pattern allowed for the maximum utilization of the surface while preventing unnecessary settlement of cells between microwells. The process of cell aggregation was directly observed from day 1, the day of cell seeding, to day 5 (Figure 3f). Both types of liver cancer cells (HepG2 and Huh7) could compactly associate, forming spheroids as integral cell clumps after 4 days of culture. MCTSs were also stained with a live/dead marker to determine their viabilities at cell seeding densities ranging from 50 to 200 cells/microwell at 50 cells/microwell intervals (Figure 3g). The results suggested that liver MCTSs remained viable after being cultured in the microwells for 4 days. The generated MCTSs had controllable sizes ranging from 100 to 140 μm for HepG2 cells and from 90 to 120 μm for Huh7 cells.

### 3.3. Glass Microaperture for Trapping/Positioning of Cellular Spheroids

**Figure 4:**
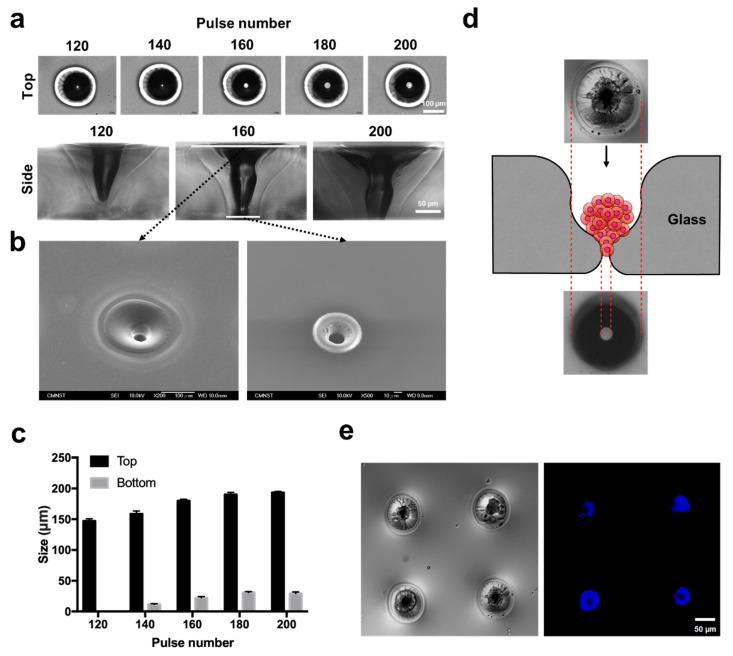
Microapertures formed by CO_2_ laser drilling on coverglass and trapping of MCTSs. (**a**) Morphologies of glass apertures with different diameters with respect to pulse numbers ranging from 120–180. (**b**) Top and bottom views of SEM images of glass apertures drilled at a pulse number of 160. (**c**) Quantification of the diameters of top and bottom glass apertures with respect to a range of pulse numbers. (**d**) Schematic illustration of MCTS trapping by a glass aperture. (**e**) Huh7 MCTSs trapped at the neck of the hourglass shape; differential interference contrast (left) and fluorescence images of nuclei (Hoechst staining).

Similar laser drilling parameters applied to conventional coverglass can result in a through-hole microaperture applicable for cell trapping (Figure 4). To identify the required power for proper perforation of the coverglass, different laser pulse numbers were investigated (Figure 4a). At pulses of 120 and 140, no distinct aperture was found via the side view; however, an enlarged through-hole was gradually observed by varying the laser pulses from 160 to 200 at 20 pulse increments. The top and bottom views of apertures ablated at a pulse of 160 were further examined by SEM, showing smooth and debris-free surfaces (Figure 4b). The diameter of the perforated microaperture ranged from 15 to 20 μm for the pulse condition tested (Figure 4c). Given that the microaperture inherently exhibited an hourglass morphology after laser ablation, the concaved region was utilized for trapping cells/cellular spheroids when negative suction was applied (Figure 4d). A 2 × 2 microaperture array was able to trap Huh7 MCTSs labeled with Hoechst (nuclei), indicating that cellular contents can be positioned based on the array design (Figure 4e).

### 3.4. PS Microapertures for Hanging Drop-Based Drug Screening

**Figure 5:**
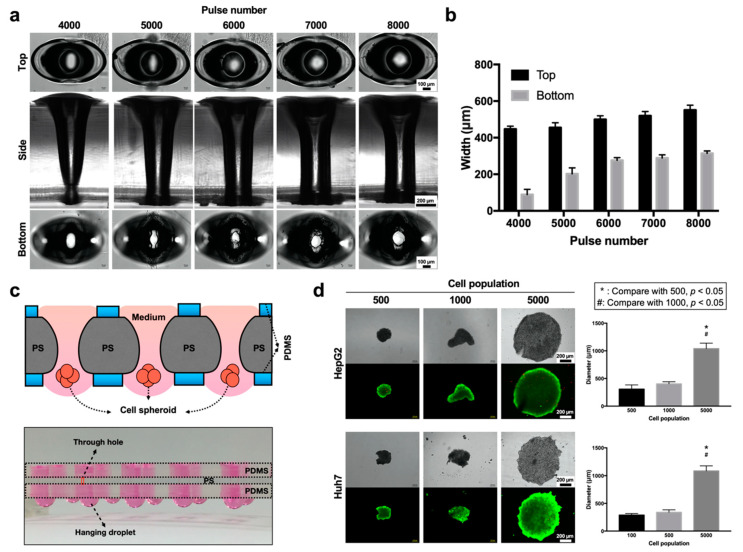
Characterizations of PS through-hole structures for the formation of Huh7 and HepG2 MCTSs. (**a**) Images of through-hole ablation at various angles (top, cross section, bottom). (**b**) Evaluation of the top and bottom widths of both sides of the through-hole structure at different pulse numbers. (**c**) Illustration of the designed 10 hanging drop spheroid culture array device (top) and its cross-sectional view (bottom). (**d**) Bright-field (gray) and fluorescence images of live (green) /dead (red) stained HepG2 and Huh7 cell spheroids (5000 cell populations) generated with various starting cell populations over a 4-day culture period (left). Average diameters of HepG2 and Huh7 spheroids over the 4 days of culture using various initial cell numbers per spheroid (right).

By increasing the pulses to perforate the PS substrate, we demonstrated that laser drilling can further enable convenient fluid exchange during the growth of MCTSs via the hanging drop method (Figure 5). To ensure complete penetration of the PS substrate, laser pulses were applied from 4000 to 8000 at 1000 pulse increments (Figure 5a). In the top view, the morphology of the axisymmetric wing protrusions is shown and is similar to that of the structures observed during microwell fabrication. From the side view, all parameters resulted in perforated PS substrates and created an open interior channel, with the edge of the through-hole becoming straighter as the pulse number was increased. A slightly irregular opening was observed at the bottom of the through-hole at pulse numbers ranging from 5000 to 7000. The through-hole at a pulse number of 8000 had a circular opening that was utilized for additional hanging drop application. Size measurements of all the fabricated parameters were performed on the top and bottom of the through-hole channel (Figure 5b). The top of the through-hole measured between 440 and 550 μm, and the bottom through-hole measured between 100 and 300 μm. A schematic diagram shows that the perforated PS sheet was sandwiched between two PDMS slabs to facilitate liquid handling and the formation of spheroids using the hanging drop technique. The picture depicts the assembly of the hanging drop chip that contained 10 drops of spheroids cultured under array conditions (Figure 5c). The design permits direct fluid exchange via a micropipette without affecting the spheroid. Time-lapsed images showed the process of cell aggregation from day 1, the day of cell seeding, to day 5 within the hanging droplet. Live/dead staining was used to assess the cell viability of the MCTSs formed in the hanging drop on day 5 over seeding concentrations of 500, 1000 and 5000 cells droplet^−1^ (Figure 5d). Our results suggested that the MCTSs within both Huh7 and HepG2 cells exhibited good viability, but the shapes of the spheroids varied widely among the different concentrations. An evaluation of the relationship between MCTS size and cell number indicated that the hanging drop method could generate spheroids ranging from 300 μm to 1000 μm in diameter at desired seeding concentrations.

**Figure 6:**
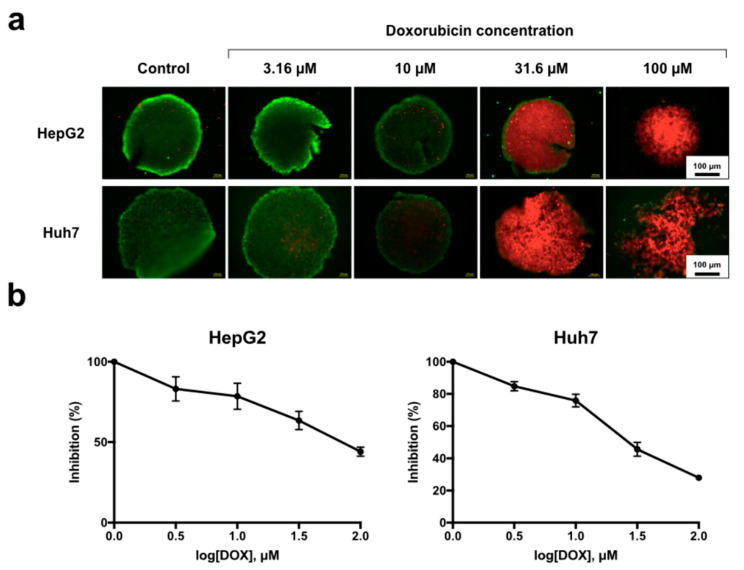
Live/dead viability and WST-1 assays after Doxorubicin hydrochloride (DOX) treatment. (**a**) Live (green)/dead (red) stained images of 5000 cell HepG2 and Huh7 spheroid cells viabilities at various DOX concentrations at 24 h after drug treatment. (**b**) Measurement of the cytotoxicity on DOX to HepG2 and Huh7 spheroids using the WST-1 cell proliferation assay.

The MCTSs were subjected to live/dead viability and WST-1 assays after DOX drug treatment (Figure 6). The morphologies and viabilities of the spheroids after 24 h of treatment with DOX at desired concentrations are shown (Figure 6a). Under conditions of 10, 31.6 and 100 μM of DOX treatment, red fluorescence was visibly observed within the spheroids, suggesting that a detrimental effect was achieved in both types of hepatocellular carcinoma cells. Dissociation of the spheroids was observed for Huh7 cells in the 100 μM DOX treatment group. The viabilities of both hepatocellular carcinoma spheroids were quantitatively assessed using the WST-1 assay (Figure 6b). Both cell lines showed dose–response behaviors to drug treatment, with the HepG2 spheroids displaying more drug resistance than the Huh7 spheroids.

## 4. Discussion

MNs can easily penetrate through the epidermis to deliver a broad range of molecules, especially macromolecules, into the skin without causing notable pain. Therefore, MNs have been considered as a convenient, safe, and effective system for transdermal drug delivery. Compared to MNs made of silicon and metals, polymeric MNs have attracted enormous attention because many polymers are biocompatible, biodegradable and nontoxic. Polymeric MNs can be fabricated on a large scale and can encapsulate large amounts of the drug [19]. Additionally, polymers with different physical and chemical properties and degradation behaviors can be utilized to fabricate MNs with different drug release profiles for a variety of disease treatments.

Polymeric MNs are commonly made using a micromolding technique, which involves fabrication of a master MN structure, creation of a female PDMS mold from the master structure, and casting of polymers into the PDMS mold [20]. A number of methods have been utilized to produce these master MN structures, such as the microelectromechanical systems (MEMS) technique for silicon-based structures and the electrodischarge machining process for metallic-based structures [21]. However, these techniques are usually complicated and time-consuming, and require expensive equipment or cleanroom conditions. Nejad et al. previously investigated a high-aspect-ratio MN mold using CO_2_ laser engraving on acrylic sheets [22]. In this study, we demonstrated that PDMS molds with various shapes and geometries could be easily fabricated using the CO_2_ laser ablation technique and adjusting the FPP and laser pulse number (Figure 2). After tuning the laser parameters, PDMS molds with widths ranging from 250 to 700 μm and depths ranging from 150 to 1400 μm were generated (Figure 2a,b). The obtained female PDMS molds can be directly used to fabricate polymeric MNs without the need for a master MN structure, thus eliminating many complicated steps and expensive cleanroom facilities.

A variety of polymeric MN shapes with different heights and tip sharpness were successfully prepared by casting the PVP/PVA solution into the laser-ablated mold (Figure 2d,e). Both PVP and PVA are water-soluble polymers and have been widely used in biomedical applications due to their biocompatibility, biodegradability, and nontoxic nature [23,24,25]. Additionally, these polymers have been approved by the FDA for clinical uses in humans [24,26]. Thus, PVP/PVA was used as a representative example to fabricate biodegradable MN arrays for proof of concept. We showed that the prepared drug-loaded PVP/PVA MNs had sufficient mechanical strength to be inserted into a porcine skin and that they were able to deliver the loaded macromolecular drugs into the skin (Figure 2g). These results demonstrate the feasibility of using the proposed technique to fabricate biodegradable polymer-based MNs for transdermal drug delivery. This laser ablation approach enables the facile, on-demand and cleanroom-free fabrication of MN molds with the desired geometry, thus allowing mass production of MNs in a cost-effective way.

Rapid CO_2_ laser prototyping has been previously demonstrated as an effective alternative for the fabrication of microwells for the formation of size-controlled 3D spheroids [3,11,13,16]. However, one major obstacle that has remained non-demonstrated in PS microwells is the inevitable loss of cells after cell seeding because of the required washing step to ensure that cells are not lodged into the microwells and can be efficiently removed without perturbing the size-uniformity of the MCTSs [27]. To overcome this issue, the design of the microwell array arrangement should be as compact as possible to allow for complete surface utilization. Although PS microwells inherently possess a pair of wing recast structures due to the heat–melt process, the space between each microwell could be maintained with a marginal thickness and the formation of a barrier between microwells for the compartmentalization of the MCTSs (Figure 3). Our results demonstrated that an improved microwell array arrangement could be utilized to yield uniform Huh7 and HepG2 MCTSs without an additional washing step after cell seeding. Additionally, choosing PS as the microwell substrate is favorable from a biologist’s perspective because PS is the most commonly utilized plastic for in vitro cell culture research. Compared to PDMS [28] or polyester [13] microwells, PS is hassle-free for the researchers in terms of the material accessibility, and more amenable in terms of compatibility with different forms of conventional culture plasticwares, such as Petri dishes [3] or microtiter plates [16]. In addition, the PS microwell displayed a gentle concave bottom, and curved microwell structures have been reported to promote the aggregation of spheroids [29]. The exact time required for the formation of MCTS as well as the specific MCTS morphology is highly dependent on the cell number, microwell dimension and cell line [30]. From our observation, initial fibroblast aggregation (data not shown) occurred rapidly after one day (on day 2) of cell seeding, which was also observed in a similar study [31]. However, a similar aggregative morphology was observed for only HepG2 cells on day 3 and for Huh7 cells on day 4 (Figure 3f).

In addition to generating a concave structure on the substrate, as demonstrated in the previous sections, penetrated microapertures can also result in several expanded utilizations. We demonstrated that a microaperture was conveniently generated on a conventional coverglass (Figure 4). Given that CO_2_ laser drilling provided an intense local heat source, the glass substrate reached the glass transition temperature and started to melt and flow to form an hourglass-shaped aperture with a size of ≈15 µm [10]. By introducing two-stage laser drilling, the perforated aperture reached 1–3 µm, which is suitable for electrophysiological ion channel recordings [10]. We previously demonstrated that the ablated hourglass-shaped glass aperture could be integrated with microfluidic channels for solution exchange, patch-clamp recordings and drug discovery [17]. A few months ago, Ayan et al. showed that spheroid aspiration could assist in bioprinting control [32]. Herein, the pattern of a microaperture array demonstrated that parallel spheroid trapping is possible for precise positioning.

The hanging drop method, which employs spheroid aggregation in a culture medium drop based on the drop shape and gravity, is one of the most commonly used methods for generating 3D models with minimal requirements for additional equipment [33,34,35]. Although the typical hanging drop technique can be performed on the underside of culture plate lids, one of the major limitations of this technique is the difficultly in media exchange [36]. Several commercial products and lab-developed platforms have included a microchannel or incorporated microfluidics for ease of liquid handling [37,38,39]. Such modifications allow the media that spheroids are formed to be replaced by fresh media for better metabolic and intertissue communication [39]. However, the implementation of clean-room microfabrication has again become inevitable. Additionally, high-throughput screening may not be necessary for preliminary lab investigations in which only a few data points a day can be sufficient. In this study, we fabricated uniform through-hole structures with a CO_2_ laser, and we could easily generate uniformly shaped drops within the same drop volume (Figure 5). In addition, direct media exchange by micropipetting was also accomplished without affecting the spheroids for studying the drug efficacy of DOX in HepG2 and Huh7 liver MCTSs (Figure 6).

Finally, CO_2_ laser-assisted microfabrication has been a preferred method for rapid in-house prototyping and manufacturing in the laboratory because of its cost-effectiveness and pattern flexibility as well as the ease of system accessibility [3,10,17]. Several notable studies have demonstrated its feasibility for potential uses in biomedicine, such as in biochips and microfluidic devices for cellular-based assays [40,41]. Among various applications enabled by the CO_2_ lasers, this work explicitly focused on providing an integrated viewpoint of single point-drilling in the generation a microstructure array. The results not only serve as a reference for researchers with different downstream applications but also provide a step-by-step workflow to identify potentially new structures in different materials. For instance, for researchers interested in the microstructure of laser-ablated PMMA (Figure S2), the laser pulse parameters, FPP distance and methods for microstructure examination can all be determined based on the current work. This outcome, again, highlights that the seemingly disparate downstream applications of MN molds, microwells and microapertures were all derived from an identical protocol and unified parameters that involve minimal changes to the materials used. This work hopefully serves as a holistic guide for CO_2_ laser microfabrication in regards to laser protocols, material choices and potential utilities to study biomedicine form a wide range of perspectives.

## 5. Conclusions

Protocols were developed to provide an extremely versatile method of CO_2_ laser drilling for the rapid prototyping of various microstructures with different substrate materials in an attempt to showcase the wide usability range of the proposed method. This point-ablation method could directly generate different types of MN molds with PDMS substrates by simply varying the FPPs and pulse numbers. The same method applied to a PS substrate resulted in the formation of a compact microwell structure that could be used to produce cell loss-free and size-controlled MCTSs. The fabrication of a through-hole in different substrates was demonstrated in which a coverglass yielded a microaperture suitable for the positioning of MCTSs and a PS microaperture could facilitate media exchange in the hanging drop method. The formation and drug screening of MCTSs in the hanging drop method confirmed the feasibility of using this method for drug interrogation.

## Supplementary Materials

The following are available online at https://www.mdpi.com/2227-9059/8/10/427/s1.
