# Supplementary material for: A Facile Approach for Rapid Prototyping of Microneedle Molds, Microwells and Micro-Through-Holes in Various Substrate Materials Using CO2 Laser Drilling
Source: Biomedicines. 2020 Oct 18;8(10):427. doi: 10.3390/biomedicines8100427 (PMC7603185; doi:10.3390/biomedicines8100427)
Supplement: Supplementary file 1 [file biomedicines-08-00427-s001.pdf]

biomedicines

MDPI

Article

A Facile Approach for Rapid Prototyping of Microneedle Molds Microwells and Micro-Through-Holes in Various Substrate Materials Using CO2 Laser Drilling

Yu-Wei Chen[1], Prof. Mei-Chin Chen [2], Kuang-Wei Wu [1], Prof. Ting-Yuan Tu [1,3,4*]

[1] Department of Biomedical Engineering, National Cheng Kung University, Tainan 70101, Taiwan; edfu237bc@gmail.com (Y.-W.C.); stephen0001345@gmail.com (K.-W.W.)

[2] Department of Chemical Engineering, National Cheng Kung University, Tainan 70101, Taiwan; kokola@mail.ncku.edu.tw

[3] Medical Device Innovation Center, National Cheng Kung University, Tainan 70101, Taiwan

[4] International Center for Wound Repair and Regeneration, National Cheng Kung University, Tainan 70101, Taiwan

* Correspondence: tingyuan@mail.ncku.edu.tw; Tel.: +886-966-570-573

Received: 26 September 2020; Accepted: 15 October 2020; Published: date

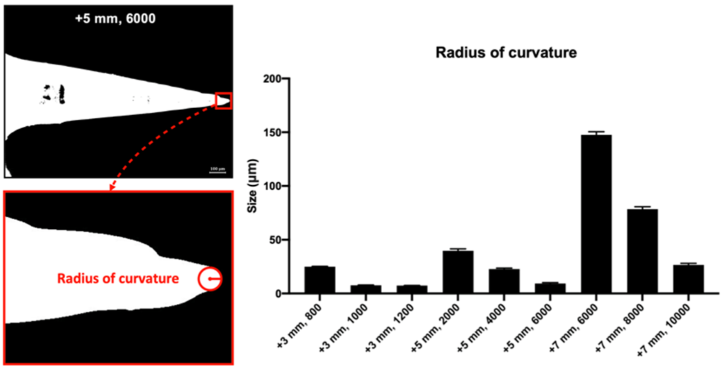

Figure S1. Measurement of the radius of curvature of the PVP/PVA MNs. The left images show the measurement of the radius of curvature. The right figure shows the size of the radius of curvature of the PVP/PVA MNs created with various CO_2 laser pulse numbers in FPPs.

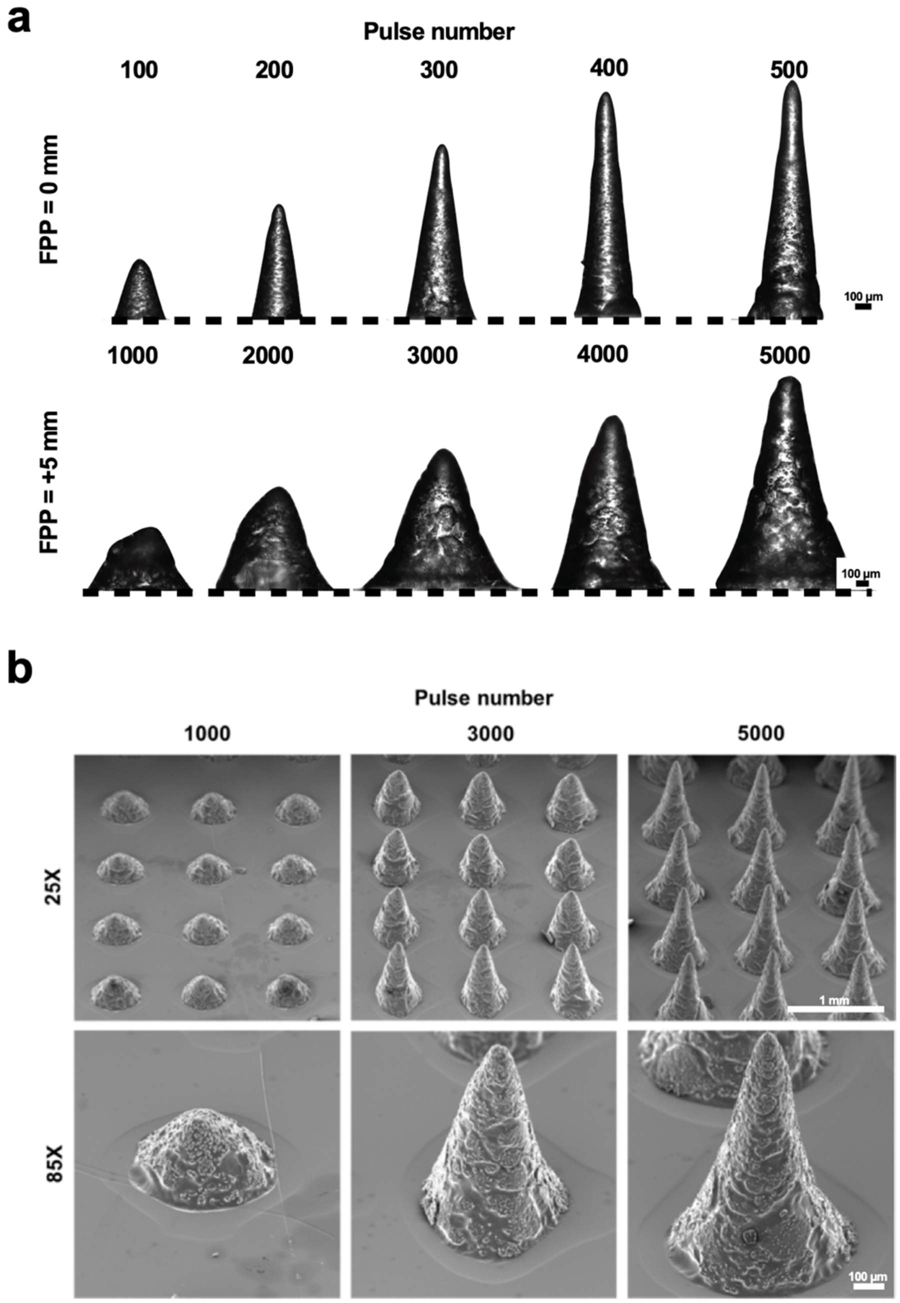

Figure S2. Example of a microstructure identification using a laser-ablated PMMA mold. (a) Side view of the PMMA molds created with different pulse numbers (1000-5000) and FPPs (0 and +5 mm). (b) SEM images of PDMS MN array replica molded from the PMMA molds.
